# MglB Fills a GAP in Bacterial Polarity and Motility

**DOI:** 10.1371/journal.pbio.1000431

**Published:** 2010-07-20

**Authors:** Caitlin Sedwick

**Affiliations:** Freelance Science Writer, San Diego, California, United States of America

**Figure pbio-1000431-g001:**
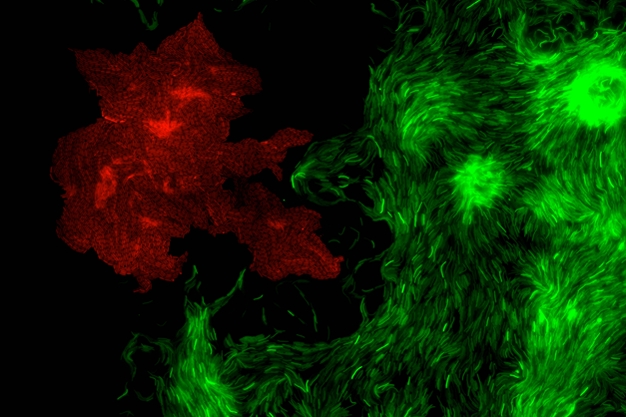
Predation of an *Escherichia coli* microcolony (red) by a *Myxococcus* “wolf pack” (green). Control of directed motility by the small GTPase MglA is essential for this highly concerted multicellular behavior. (Image: Adrien Ducret).

[Fig pbio-1000431-g001]Bacteria exemplify—in miniature and simplified form—many of the processes found in more complex, eukaryotic cells. For example, like their eukaryotic cousins, motile bacteria exhibit polarized morphologies: their front and back ends are distinguished by different concentrations of proteins and specialized cellular structures like pili or flagella. And, just as motile eukaryotic cells can reorganize the proteins at their peripheries to effect a change in direction, at least one bacterium—the soil bacterium *Myxococcus xanthus*—can reorganize the proteins at its front and rear ends to reverse its direction of motion.

*M. xanthus* is a predator that hunts for prey bacteria using a random walk pattern. While on the move, the rod-shaped bacterium crawls along its long axis using one of two motility mechanisms—S-motility and A-motility. In S-motility, pili extruded from the front end of the cell are used like grappling hooks to drag the cell forward. Meanwhile, in A-motility, adhesion complexes constructed at the front of the cell are ratcheted toward the rear (where they are disassembled), and the bacterium perches atop these adhesion complexes like a tank on treads to achieve forward motion. In both cases, *M. xanthus* also periodically reverses its direction of movement in order to cover more surface area while hunting. These reversals occur with a frequency that's thought to be controlled by biochemical oscillations in a group of proteins known as the Frz complex. However, how Frz complex oscillations cause these directional reversals is unknown. In this issue of *PLoS Biology*, Yong Zhang, Tâm Mignot, and colleagues demonstrate that directional reversals involve a protein apparatus strikingly reminiscent of the one eukaryotic cells use.

In eukaryotic cells, cellular polarity and motility are controlled by small GTPases (e.g., Ras, Rac, and others). In their GTP-bound state, these proteins can bind to and activate other protein targets, some of which directly modify the cell cytoskeleton, and others that participate in crosstalk with other GTPases. However, after GTP hydrolysis (prompted by a class of accessory proteins known as GTPase activating proteins, or GAPs), GTPases adopt an inactive, GDP-bound conformation. Modulation of the GTPase cycle is therefore critical to the regulation of cellular polarity and motility.

As with eukaryotic cells, a small GTPase—the Ras-like protein MglA—has long been known to play a critical role in *M. xanthus* cellular polarity and motility. MglA localizes to the front end of migrating cells, and when cells reverse directions, it is always found at the new front end. There, it controls the localization of other proteins needed for both A- and S-motility. But what controls MglA localization?

Zhang and colleagues hypothesized that MglA localization and activity might be controlled by MglB, a protein of heretofore unknown function that is always co-expressed with MglA. Bioinformatics studies suggested MglB might modulate MglA GTPase activity, so the group tested whether MglB affects MglA's GTP hydrolysis rate in vitro. Indeed, they found that MglA significantly accelerates GTP hydrolysis by MglA. This suggests that MglB is a GAP for MglA, serving to switch MglA to its inactive, GDP-bound state.

Fluorescent tagging of MglB and MglA in live bacteria revealed that MglA and MglB are always at opposite ends of migrating cells, with MglA at the front and MglB at the back. When cells switch direction, MglA and MglB swap position, too, leading the authors to suggest that MglB restricts GTP-bound, active MglA to the front of the cell.

Consistent with this idea, Zhang et al. found that when MglB is deleted from cells, MglA is mislocalized—it accumulates at both poles. However, these MglB-deficient cells reverse direction much more frequently than wild-type cells. At first blush, this is rather counterintuitive; if MglA determines the front of the cell, how are MglB-deficient cells, where MglA is at both ends of the cell, able to reverse at all, much less do so more frequently than normal? The authors explain that these reversals come about thanks to a quirk of A-motility: GTP-bound MglA associates with A-motility focal adhesions, and as a result, is continually being trafficked toward the rear of the cell. In the absence of MglB, GTP-bound MglA accumulates at the rear until it is at sufficient concentration to define a new “front.”

Zhang and colleagues went on to demonstrate that MglB acts under the control of the Frz pathway to regulate the GTP-bound state of MglA. The discovery of MglB as the first bacterial GAP, and the elucidation of the roles MglB and MglA play in regulating cellular polarity and motility, shows that these proteins are an exemplar for a critical, evolutionarily conserved pathway.

**Zhang Y, Franco M, Ducret A, Mignot T (2010) A Bacterial Ras-Like Small GTP-Binding Protein and Its Cognate GAP Establish a Dynamic Spatial Polarity Axis to Control Directed Motility. doi:10.1371/journal.pbio.1000430**

